# Preclinical evaluation of a GFRA1 targeted antibody-drug conjugate in breast cancer

**DOI:** 10.18632/oncotarget.25160

**Published:** 2018-05-01

**Authors:** Emily E. Bosco, R. James Christie, Rosa Carrasco, Darrin Sabol, Jiping Zha, Karma DaCosta, Lee Brown, Maureen Kennedy, John Meekin, Sandrina Phipps, Joanne Ayriss, Qun Du, Binyam Bezabeh, Partha Chowdhury, Shannon Breen, Cui Chen, Molly Reed, MaryJane Hinrichs, Haihong Zhong, Zhan Xiao, Rakesh Dixit, Ronald Herbst, David A. Tice

**Affiliations:** ^1^ Oncology Research, MedImmune, LLC, Gaithersburg, Maryland, United States of America; ^2^ Antibody Discovery and Protein Engineering, MedImmune, LLC, Gaithersburg, Maryland, United States of America; ^3^ Pathology, MedImmune, LLC, Gaithersburg, Maryland, United States of America; ^4^ Pathology, MedImmune, Ltd, Cambridge, United Kingdom; ^5^ Biologics Safety Assessment, MedImmune, LLC, Gaithersburg, Maryland, United States of America; ^6^ Department of Global Biotherapeutics, Pfizer, Cambridge, Massachusetts, United States of America; ^7^ Research, Salubris Biotherapeutics, Gaithersburg, Maryland, United States of America; ^8^ Translational Sciences, NGM Biopharmaceuticals, South San Francisco, California, United States of America; ^9^ Biologics Discovery, Sanofi Genzyme, Cambridge, MA, United States of America

**Keywords:** GFRA1, antibody-drug conjugate (ADC), pyrrolobenzodiazepine (PBD), anti-tumor activity, breast cancer

## Abstract

Despite recent advances in treatment, breast cancer remains the second-most common cause of cancer death among American women. A greater understanding of the molecular characteristics of breast tumors could ultimately lead to improved tumor-targeted treatment options, particularly for subsets of breast cancer patients with unmet needs. Using an unbiased genomics approach to uncover membrane-localized tumor-associated antigens (TAAs), we have identified glial cell line derived neurotrophic factor (GDNF) family receptor α 1 (GFRA1) as a breast cancer TAA. Immunohistochemistry (IHC) revealed that GFRA1 displays a limited normal tissue expression profile coupled with overexpression in specific breast cancer subsets. The cell surface localization as determined by fluorescence-activated cell sorting (FACS) and the rapid internalization kinetics of GFRA1 makes it an ideal target for therapeutic exploitation as an antibody-drug conjugate (ADC). Here, we describe the development of a pyrrolobenzodiazepine (PBD)-armed, GFRA1-targeted ADC that demonstrates cytotoxicity in GFRA1-positive cell lines and patient-derived xenograft (PDX) models. The safety profile of the rat cross-reactive GFRA1-PBD was assessed in a rat toxicology study to find transient cellularity reductions in the bone marrow and peripheral blood, consistent with known off-target effects of PBD ADC’s. These studies reveal no evidence of on-target toxicity and support further evaluation of GFRA1-PBD in GFRA1-positive tumors.

## INTRODUCTION

Approximately 70% of breast tumors are estrogen receptor α (ER) positive and thus amenable to endocrine-disrupting therapies. However, numerous mechanisms of de novo or acquired resistance to endocrine therapy lead to disease recurrence and metastases in 30% of patients with ER-positive cancers [1, 2]. Likewise, because there are no targeted therapies for triple-negative breast cancer (TNBC), patients must rely on standard chemotherapeutic regimens that are associated with high rates of local and distant relapse [3]. Thus, despite the many therapeutic successes in breast cancer, novel therapies are still needed for large subsets of patients. A greater understanding of the shared molecular characteristics of breast tumors could guide the development of optimal tumor-targeted therapeutic interventions.

Over the past several years, the antibody-drug conjugate (ADC) has emerged as a therapeutic platform that can exploit tumor-specific molecular characteristics. ADCs comprise a cytotoxic drug chemically attached to a tumor-specific antibody, to increase the amount of drug targeted to the tumor. Ado-trastuzumab emtansine, which debuted in 2013 for the treatment of HER2-positive breast cancers, is one of the most notable ADCs that has demonstrated clinical success [4]. Otherwise, ADCs have had limited applicability in breast cancer to date. One reason for limited success involves extremely potent payloads that can induce off-target toxicities before reaching therapeutic dose levels in Phase I clinical trials [5]. Another reason is the narrow therapeutic index of many ADC programs, which arises from the relative scarcity of tumor antigens that are overexpressed in tumor tissues but not in essential normal tissues. Identifying tumor-associated antigens (TAAs) with exceptionally limited expression in critical normal tissues can help in overcoming these problems.

The breast cancer antigen, GFRA1, is a 51-kDa glycosylphosphatidylinositol (GPI)-linked cell-surface receptor for GDNF and coactivator of RET [6–8]. Canonically, the GDNF/GFRA1 complex activates RET to potentiate downstream signaling through the mitogen-activated protein kinase (MAPK)/extracellular-signal-regulated kinase (ERK) and phosphoinositide 3-kinase (PI3K) pathways, promoting the differentiation, proliferation, and survival of neurons. Non-canonical GFRA1 signaling, which is independent of RET and may operate through the L1 and neural cell adhesion molecules (L1CAM, NCAM), among others, is poorly understood [9].

GFRA1 is not expressed in adult tissues, except for the mammary glands, hair follicles, and neuronal tissues [10, 11]. In contrast, GFRA1 is overexpressed in the majority of breast cancers [12–14]. The GFRA1 axis is reported to promote breast cancer proliferation and invasion, and its expression correlates with lymph node metastases and advanced clinical stage [14–16]. GFRA1 positivity also predicts reduced overall survival and poor response to multiple modes of therapy [15]. Furthermore, GFRA1 is more prevalent and highly expressed in tumors that have become refractory to chemotherapeutics [14], and expression of this signaling pathway can facilitate resistance to aromatase inhibitors used in breast cancer therapy [17, 18]. Thus, GFRA1 could serve as a TAA for ADC targeting in breast cancers that require alternative therapeutic strategies.

Similar to recent work described by Bhakta et al. [19], here we report additional characterization of GFRA1 expression and the preclinical development of a novel ADC targeting GFRA1. We describe GFRA1 as a TAA amenable to ADC targeting due to its internalization capacity, its highly specific expression in tumor-cell membranes, and its limited expression in essential normal tissues. Uniquely, we have generated an anti-GFRA1 antibody conjugated to a PBD payload. The *in vitro* and *in vivo* activity of this ADC was explored in target-positive cell lines and extended into patient-derived xenograft (PDX) models to elucidate the target expression threshold required for ADC activity. Finally, we examined the preclinical toxicity of GFRA1-PBD in rats to characterize the safety profile and investigate potential on-target toxicity.

## RESULTS

### The *GFRA1* gene is expressed in breast cancer

**Figure 1:**
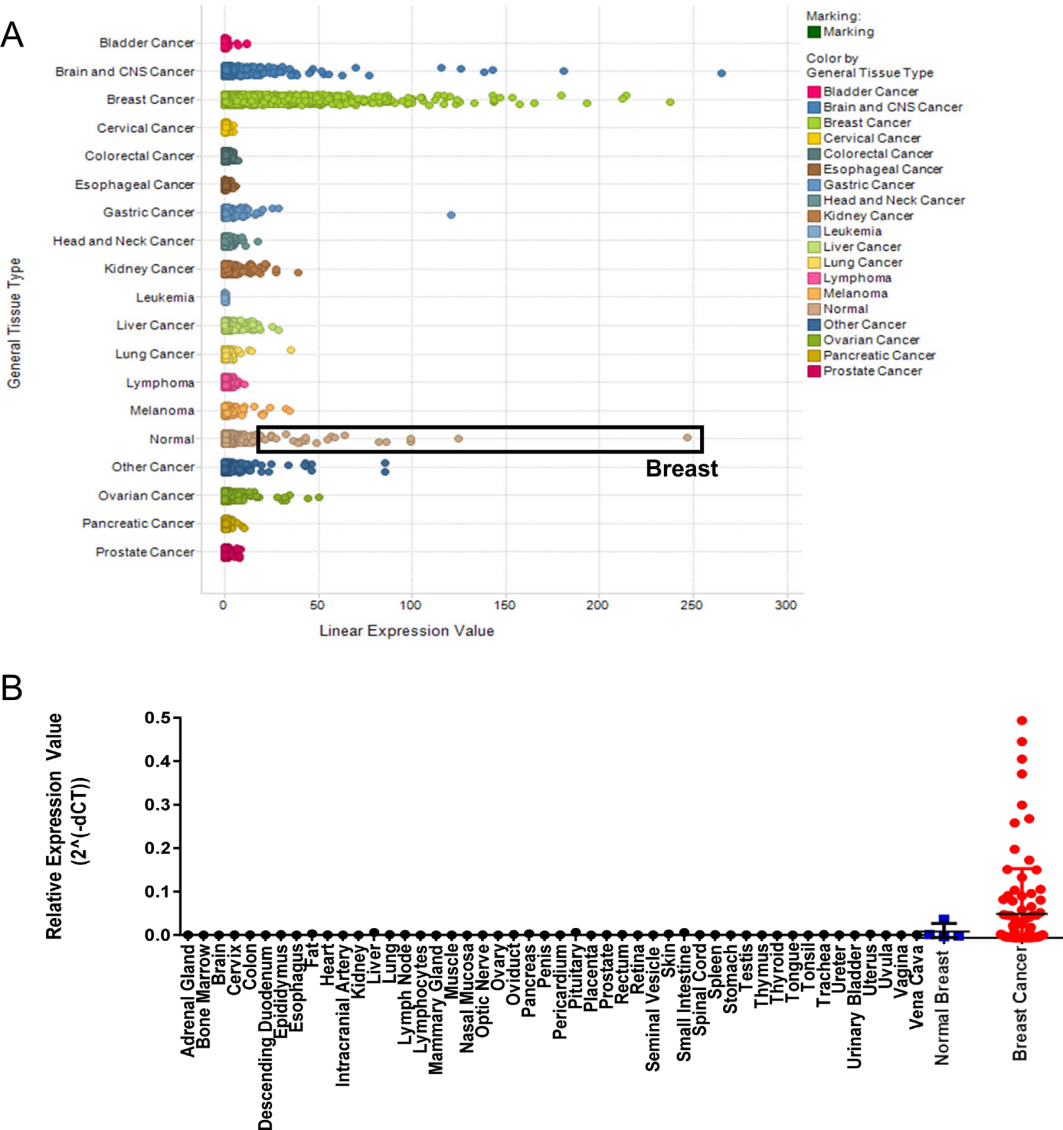
*GFRA1* is highly expressed in breast cancer (**A**) Across a panel of human cancer samples, *GFRA1* expression is highest in breast cancer. Normal tissue samples exhibiting the highest *GFRA1* expression were from breast tissue, defined by the black box. Analyses were done by using the Oncomine Power Tools database (powertools.oncomine.com). (**B**) *GFRA1* expression was highest in normal breast tissue and in breast cancers, as determined by quantitative real-time PCR analysis of cDNA arrays from Origene.

We used the Oncomine Power Tools gene expression database to interrogate the expression of *GFRA1* across multiple major cancer types and distal normal tissues. Consistent with other studies, *GFRA1* expression was highest in normal breast tissue, compared with other normal tissues. *GFRA1* was also expressed highly in breast cancer tissue, while other tumor types did not exhibit significant expression (Figure 1A). cDNA array gene expression profiling of a normal human cDNA array and two different breast cancer arrays confirmed the significant overexpression of *GFRA1* in breast cancers and limited expression in normal tissues (Figure 1B).

### Generation and characterization of antibodies

**Figure 2:**
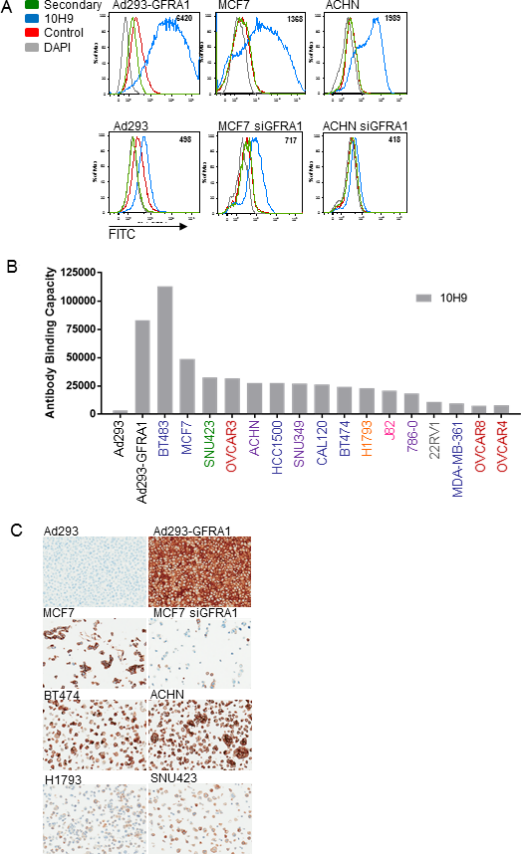
Anti-GFRA1 antibodies demonstrate specificity in model systems (**A**) Demonstration of anti-GFRA1 mAb 10H9 specificity by FACS binding to target-positive and target-negative model systems. (**B**) Evaluation of GFRA1 receptor density on the cell surface of cancer cell lines using antibody 10H9. Tissue of origin of each line is defined by color coding of of their names: embryonic kidney = black, mammary carcinoma = blue, hepatocellular carcinoma = green, ovarian carcinoma = red, renal cell carcinoma = purple, lung adenocarcinoma = orange, bladder carcinoma = pink, prostate carcinoma = grey. (**C**) IHC specificity of the anti-GFRA1 mAb 4D12. FACS, fluorescence-activated cell sorting; IHC, immunohistochemistry; mAb, monoclonal antibody.

An antibody generation hybridoma campaign yielded a panel of four high-affinity monoclonal antibodies—4D12, 9B3, 10H9, and 18B2— which bound to human, mouse, and rat GFRA1 recombinant protein, as measured by Octet (Supplementary Table 1). Antibody specificity was demonstrated by FACS using the anti-GFRA1 clone 10H9 (Figure 2A). As expected, GFRA1 appeared on the cell surface in cells expressing the protein (top row, Figure 2A), and that expression was diminished in GFRA1-null or siRNA-treated cells (bottom row, Figure 2A). GFRA1 cell surface receptor density was interrogated in various cancer cell lines reported to have high *GFRA1* RNA expression levels (Figure 2B). Next, GFRA1 IHC (4D12) was performed in order gain an understanding of the correlation between our IHC assay signal and GFRA1 receptor density values determined by FACS (10H9) (Figure 2C). Target specificity of the 4D12 GFRA1 clone was demonstrated in paired, isogenic GFRA1-expressing cell lines (top two rows, Figure 2C).

### GFRA1 tissue expression profile

**Figure 3:**
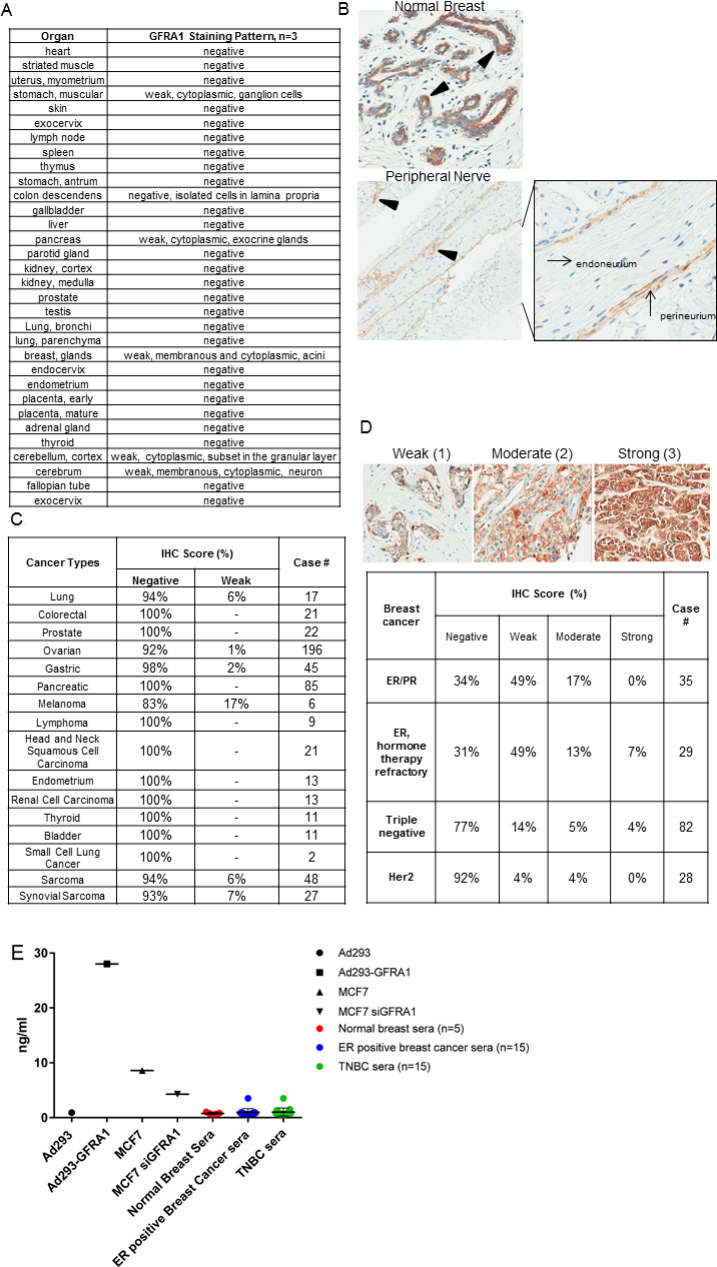
GFRA1 is a breast cancer TAA with limited expression in normal tissue (**A**) A normal-tissue microarray was subjected to IHC staining with the anti-GFRA1 mAb 4D12 to highlight membranous GFRA1 staining in breast glands and tissues from the central and peripheral nervous systems. (**B**) Representative IHC images of normal breast and peripheral nerve tissue illustrate the heterogeneous membrane and cytoplasmic localization (highlighted with triangle) of GFRA1. (**C**) IHC across a wide range of tumor types (other than breast) revealed little to no staining. (**D**) GFRA1 expression revealed multiple staining patterns across various subtypes of breast cancer (top). The prevalence of expression in each subtype is detailed in the table (bottom). (**E**) Levels of soluble GFRA1 in cell-culture models, serum from healthy donor control, and serum from patients with ER-positive breast cancer or TNBC were determined by ELISA. Anti-GFRA1 antibodies 9B3 and 18B2 were used as the capture and detection antibodies, respectively. ER, estrogen receptor; IHC, immunohistochemistry; mAb, monoclonal antibody; TNBC, triple-negative breast cancer.

The 4D12 antibody clone was also used to comprehensively study GFRA1 in a normal tissue microarray by IHC. Weak tissue staining was limited to the cytoplasmic regions of the ganglion cells in the stomach, exocrine glands in the pancreas, and a subset of cells in the granular layer of the cerebellum. Weak membrane and cytoplasmic staining patterns were also evident in the neurons of the cerebrum and in isolated cells in the lamina propria of the colon (Figure 3A). Normal breast glands, as well as the perineurial cells that support peripheral nerves, displayed a combination of weak to moderate membranous and cytoplasmic staining (Figure 3B). IHC staining patterns on multi-tumor (Figure 3C) and disease-specific tumor microarrays revealed GFRA1 expression in all breast cancer subsets (Figure 3D). However, weak membrane staining or greater was present in 66% of tumors positive for the ER and progesterone receptor, 69% of ER-positive tumors that were refractory to hormone therapy, 23% of TNBCs, and 8% of HER2 positive tumors.

Because GFRA1 is a GPI-anchored protein, proteolytic cleavage could result in its shedding from the tumor cell surface. To assess the level of soluble GFRA1 antigen in cell culture media or patient serum and thus evaluate the fitness of GFRA1 for ADC targeting, we performed a sandwich ELISA with two non-competing GFRA1 antibody clones, 9B3 and 18B2, as the capture and detection antibody, respectively. This analysis revealed high levels (28 ng/ml) of shed, soluble antigen from Ad293-GFRA1 cells, contrasted with low levels (0.9 ng/ml) from Ad293 parental cells. In agreement with the receptor density and IHC expression levels in Figure 2, the level of shed antigen from MCF7 cells was in the intermediate range (8.6 ng/ml) and was abrogated by transient transfection of GFRA1 siRNA. In an assay of serum samples from healthy donors and patients with ER-positive breast cancer or TNBC, the level of soluble GFRA1 was similarly low between serum from patients with breast cancer and that from healthy donors (Figure 3E). Although the shed antigen sink cannot be detected in breast cancer patient sera, it could be a challenge facing development of an effective GFRA1-targeted therapeutic and should be further analyzed.

### Activity of a GFRA1-targeted ADC

**Figure 4:**
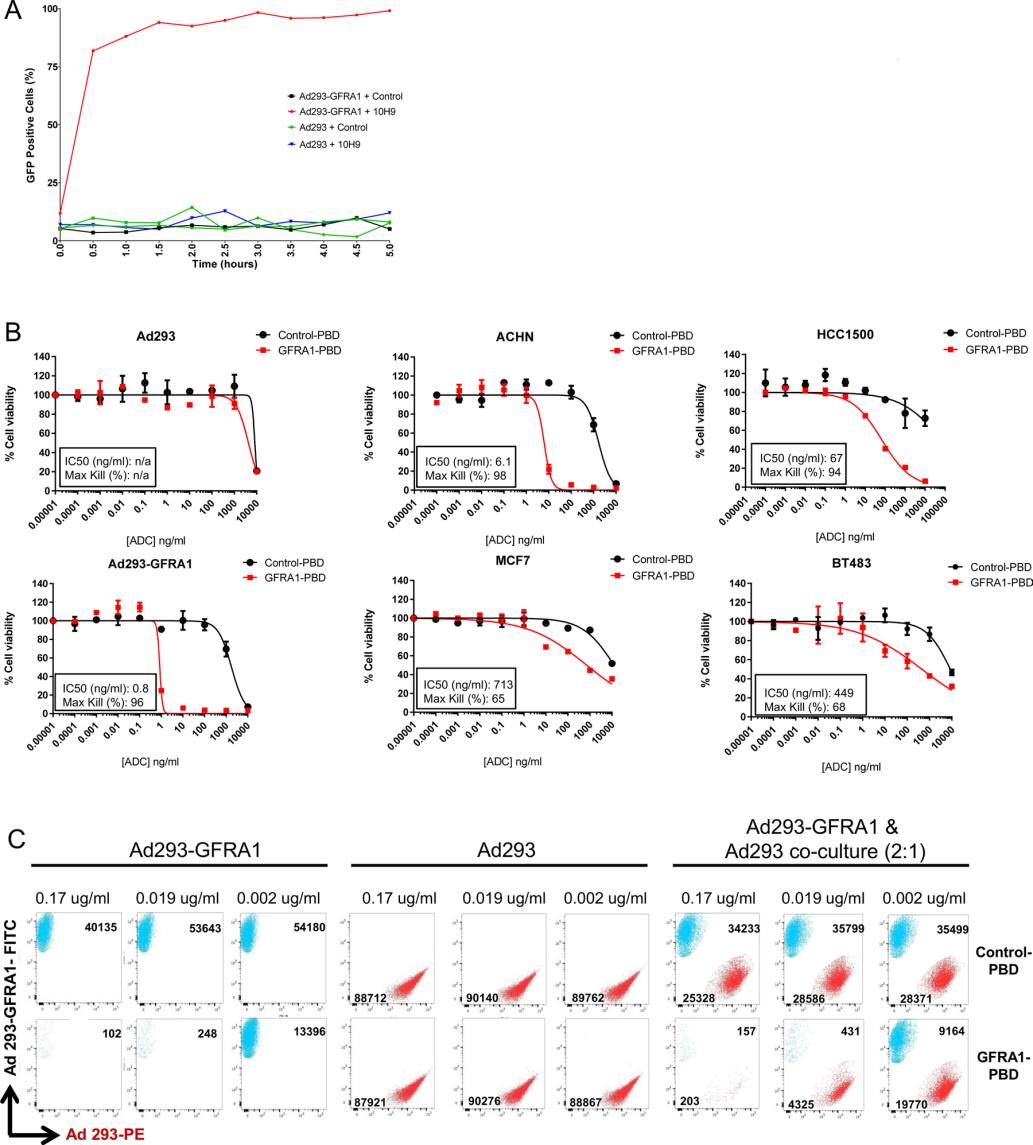
GFRA1 is a quickly internalized target suited to antibody-drug conjugate targeting (**A**) An internalization assay was performed following conjugation of 10H9 or IgG1 control with a pH-sensitive GFP dye and incubation with Ad293-GFRA1 and parental cells. Cells were imaged over a 5-hour time course to reveal the fast kinetics of 10H9 internalization and trafficking to lysosomes in target-positive cells. (**B**) *In vitro* efficacy of SG3249-conjugated 10H9 (GFRA1-PBD) was evaluated in multiple GFRA1-positive cell-line models to demonstrate target-mediated cell-killing. Representative experiments are shown and the values indicate mean + SEM. (**C**) Bystander activity of the GFRA1-PBD conjugate was assessed by FACS following 3 days of target-positive (FITC positive) and negative (PE positive) cell co-culture and ADC treatment. FACS plots for FITC and PE expression and total cell number at harvest are displayed for multiple titration points. ADC, antibody-drug conjugate; GFP, green fluorescent protein; FACS, fluorescence-activated cell sorting; FITC, fluorescein isothiocyanate; PBD; pyrrolobenzediazepine dimer; PE, proline-glutamate.

The internalization capacity of the anti-GFRA1 antibody 10H9, compared with control IgG, was assessed by conjugating the antibodies to a pH-sensitive GFP dye which only fluoresces in low pH environments (ie. endosome, lysosome) and using real-time imaging to probe internalization kinetics. At 30 minutes, more than 80% of GFRA1-positive cells were GFP positive, indicating that they had internalized the anti-GFRA1 antibody, whereas target-negative cells and cells treated with non-specific control IgG1 antibody showed no evidence of internalization (Figure 4A).

Next, antibody-drug conjugates were generated with a potent PBD payload, SG3249, and antibodies 10H9 and nonspecific control IgG1. Characterization of aggregation and drug loading of the conjugates was determined by size exclusion chromotagraphy and mass spectroscopy analysis, respectively (Supplementary Figure 1). Both conjugates consisted of > 98% monomer and displayed nearly 2 drugs loaded per antibody. The *in vitro* cytotoxicity of the 10H9-SG3249 ADC (GFRA1-PBD) was evaluated in a panel of cell lines with varying degrees of GFRA1 expression (as shown in Figure 2B, 2C). Compared with a nonspecific IgG1-SG3249 control (Control-PBD), GFRA1-PBD was cytotoxic in a range of target-positive cells, whereas only the highest dose demonstrated cell killing in target negative Ad293 cells (Figure 4B). This PBD-mediated cytotoxicity was consistent with other studies showing a dose-related increase in the intensity of IHC staining for an anti-PBD payload antibody and a γH2A.X stain indicating cellular recognition of DNA double strand breaks at 24 hours post dose (Supplementary Figure 2) [20]. Concomitant with the deposition of PBD dimer and induction of γH2A.X in ACHN cells, executioner caspases 3 and 7 were activated during the 6-day time frame of our cytotoxicity assays as determined by luminescence using Caspase-Glo^®^ 3/7 (Supplementary Figure 3). Consistent with the mechanism of action of the DNA damaging PBD warheads, this data indicates that GFRA1-PBD induces apoptosis.

In cancers with heterogenous GFRA1 expression, the antitumor activity of GFRA1-PBD hinges on the capacity of the ADC to elicit bystander activity in target-negative cells. Thus, we investigated the ability of the PBD warhead to be taken up by target-positive Ad293 cells and kill surrounding target-negative cells. Flow cytometry revealed a loss of target-negative Ad293-PE cells treated with GFRA1-PBD, but only in the presence of target-positive Ad293-GFRA1-FITC cells, indicating the existence of strong, dose-dependent bystander activity (Figure 4C).

**Figure 5:**
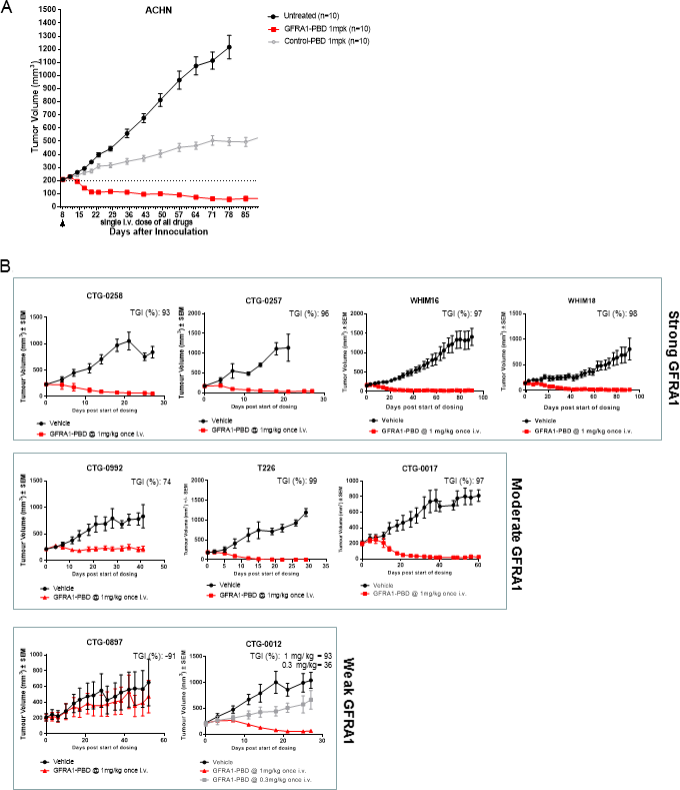
*In vivo* efficacy of GFRA1-ADC (**A**) Subcutaneous ACHN xenograft tumors were grown in athymic nude mice. When tumor growth reached 200 mm^3^, mice were randomized and treated with one intravenous 1 mg/kg dose of SG3249-10H9 (GFRA1-PBD) or SG3249-nonspecific IgG1 (Control-PBD). Tumor growth was monitored at least once per week. Representative experiments are shown and values indicate the mean + SEM, statistical significance of GFRA1-PBD treatment groups (*p* < 0.05) was demonstrated by 2-way ANOVA analysis. (**B**) PDX models were chosen and grouped based on the level of GFRA1 IHC staining of tumor slices. A single intravenous dose of GFRA1-PBD versus vehicle-control was tested in each PDX model. Tumor volume was monitored for more than 30 days. Representative experiments are shown and values indicate the mean + SEM, statistical significance of GFRA1-PBD treatment groups (*p* < 0.05) was demonstrated by 2-way ANOVA analysis for all models except CTG-0897 where *p* = 0.99. IHC, immunohistochemistry; PBD, pyrrolobenzediazepine; PDX, patient-derived xenograft.

To confirm that the *in vitro* activity evident in the cell lines expressing GFRA1 translated to *in vivo* antitumor efficacy, we assessed treatment of ACHN xenograft tumors with GFRA1-PBD or Control-PBD. Compared with untreated control and Control-PBD treated tumors, GFRA1-PBD-treated tumors showed durable regressions (Figure 5A). There is a non-specific anti-tumor response evident in Control-PBD treated animals compared to untreated controls as has been observed in other studies using PBD based ADC’s [21–23]. However, Control-PBD is significantly less active than GFRA1-PBD. No overt signs of toxicity, such as significant loss of body weight, were observed upon ADC treatment.

To better understand the *in vivo* activity of GFRA1-PBD against tumors with heterogenous levels of GFRA1 expression as seen among patients in the clinic, we assessed activity in PDX models. IHC intensity and homogeneity segregated these models into categories based on strong, moderate, or weak GFRA1 expression (Supplementary Figure 4). Compared with vehicle control, GFRA1-PBD demonstrated the greatest tumor-growth inhibition in PDX models with strong GFRA1 staining, followed by those with moderate and weak staining (Figure 5B).

Interestingly, the CTG-0012 PDX model was a TNBC stage 4 adenocarcinoma with mutations in BRCA1, ATM, BLM, and p53 (as advertised by Champions Oncology), which are involved in DNA damage repair (DDR). Evidence suggests that DDR deficiency may prime cells for hyper-sensitivity to DNA-damaging therapies [24–27]. Thus, we hypothesized that, despite the weak intensity and high heterogeneity of GFRA1 expression in CTG-0012, we might detect evidence of synthetic lethality between GFRA1 inhibition and DDR deficiency. GFRA1-PBD showed evidence of tumor inhibition at a low dose of 0.3 mg/kg and induced significant tumor regression at the standard dose of 1 mg/kg. We also assessed the activity of the combination of BRCA1 deficiency and GFRA1-PBD *in vitro* in a GFRA1-isogenic paired setting of BRCA1-proficient or deficient cells and found that the activity of GFRA1-PBD increased by nearly 15-fold in the presence of BRCA1 deficiency, compared with wild-type BRCA1 (Supplementary Figure 5).

### Toxicity of GFRA1-PBD in rats

**Figure 6:**
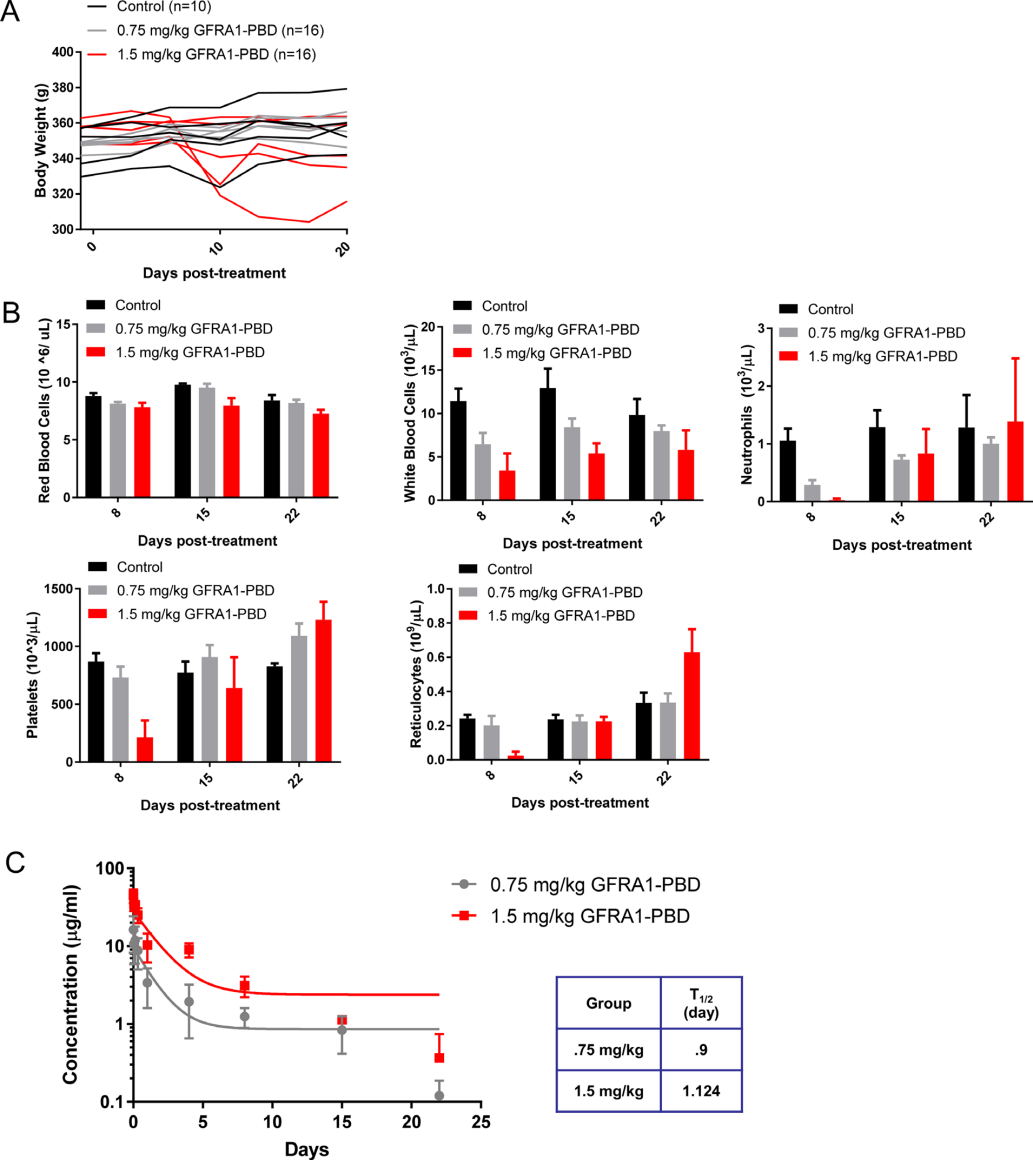
Safety evaluation of GFRA1-PBD in rat (**A**) Body weights of rats treated once with increasing concentrations of GFRA1-PBD (10H9-SG3249) or vehicle-control. (**B**) Counts of various peripheral blood cell types were obtained at Days 8, 15, and 22 following treatment with GFRA1-PBD or control. PBD, pyrrolobenzediazepine dimer. (**C**) The pharmacokinetics of the total ADC in the treated rat sera was analyzed by ELISA using an anti-PBD antibody for capture and an anti-human IgG antibody for detection. PBD, pyrrolobenzediazepine dimer.

The safety profile of GFRA1-PBD was evaluated in a non-tumor-bearing non-GLP rat toxicity model. Because GFRA1-PBD is cross-reactive with rat GFRA1, we could evaluate both on- and off- target toxicity of the molecule. At GFRA1-PBD doses up to 1.5 mg/kg, all animals survived until scheduled necropsy, with the exception of one animal from the 1.5–mg/kg-dose group that was sacrificed early because of infection related to severe myelosuppression. No significant changes in body weight were evident in any of the dosing groups (Figure 6A). We did observe a transient dose-dependent reduction in cellularity in the bone marrow and peripheral blood, which primarily affected white blood cells, neutrophils, platelets, and reticulocytes (Figure 6B). All other measured parameters and histopathology at the end of study were normal. The pharmacokinetics of the total ADC in the treated rat sera was analyzed by ELISA. To do this, an anti-PBD antibody was used to coat the plate and subsequently capture all PBD, rat sera was bound, and then an anti-human IgG antibody was utilized for detection. This data reveals a dose dependent increase in ADC in the sera with half-lives for the 0.75 and 1.5 mg/kg groups at 0.9 and 1.12 days, respectively (Figure 6C).

To understand the nature of the rat toxicity data, the normal tissue expression profile of GFRA1 in rat was confirmed by IHC in a normal tissue TMA as well as large tissue sections. In summary, the only tissues displaying positive staining were neurons in the brain, peripheral nerve ganglia as found in the colon and pancreas, as well as spermatogonia in the testis (Supplementary Figure 6). Based on this expression profile of GFRA1 in the rat, our findings confirm that the cellularity reductions seen in the bone marrow and peripheral blood upon GFRA1-PBD treatment are not on-target toxicities, but toxicities consistent with the known off-target effects of PBD-armed ADC’s [28]. Lastly, to help bridge the GFRA1 IHC expression datasets with the toxicology data and to assess the degree of GFRA1 cell surface localization, we performed FACS analysis with the GFRA1 10H9 antibody binding to human perineurial cells and rat dorsal root ganglia (DRG) (Supplementary Figure 7). The normal mammary epithelial cell line, MCF10a, was used as a negative control while MCF10a transduced with exogenous GFRA1 served as a positive control for cell surface GFRA1 expression. In this assay, the GFRA1 10H9 antibody did not bind the cell surface of MCF10a normal mammary cells or human perineurial cells despite the IHC staining of the latter (Figure 3B). Further, 10H9 displayed a low level of binding to the cell surface of rat DRG cells (Supplementary Figure 7). *In vitro* assays to determine the cytotoxicity of GFRA1-PBD in rat DRG cells were inconclusive (data not shown), perhaps due to the non-proliferative nature of these primary cells in culture. This FACS result supports the data found in Supplementary Table 1 highlighting the rat crossreactivity of the 10H9 clone, and indicates that further study to fully understand the safety profile of GFRA1-PBD is warranted.

## DISCUSSION

In this study, we demonstrate that GFRA1 may serve as an ideal TAA for ADC-targeting because of its minimal expression in normal tissue and its overexpression in subsets of therapeutically challenging breast tumor types. IHC analysis revealed that GFRA1 is expressed on tumor-cell membranes not only in ER-positive breast cancers, as has been observed in previous studies, but also in 23% of TNBCs (Figure 3D). To our knowledge, this is the first study suggesting GFRA1 is a therapeutically tractable target in a subset of TNBCs. There are no targeted therapies available for TNBC, which is associated with poor prognosis. Thus, the identification of GFRA1 and other shared TAAs with favorable tumor: normal tissue expression profiles could create additional therapeutic options for TNBC.

Our findings also suggest that GFRA1-PBD could serve as a therapeutic option for ER-positive tumors that have developed resistance to hormone therapies (Figure 3D). Previous studies have implicated the GDNF-RET signaling pathway in development of such resistance [29, 30]. IHC on breast tumors refractory to hormone therapies did not show an increase in GFRA1 expression upon therapeutic failure. Instead, the level of membrane GFRA1 expression was similar among ER-positive tumors, regardless of their sensitivity to hormone therapy. Thus, our findings uncover two breast cancer subsets that lack adequate treatment options and could respond to a GFRA1-targeted ADC.

This work has established that GFRA1 expression is associated with GFRA1-PBD activity *in vivo* in a wide range of clinically relevant PDX models (Figure 5B). As expected, we observed significant tumor growth inhibition and regression in models with strong GFRA1 expression. However, as displayed by the CTG-0012 and CTG-0017 models, many tumors positive for GFRA1 membrane expression actually display an extremely weak and heterogeneous density of GFRA1. GFRA1-PBD still showed antitumor activity in these models likely because of the high potency of the PBD warhead and the bystander activity of GFRA1-PBD. In contrast, CTG-0897 displayed high intensity, punctate patches of GFRA1 expression which was not sufficient to confer sensitivity to GFRA1-PBD. Thus, despite the potency and bystander activity of the PBD warhead, there remains a threshold of target expression required for ADC efficacy that incorporates a balance between target expression level and homogeneity across the tumor.

PBD dimers are strong DNA alkylating agents whose crosslinks lead to double-strand breaks in DNA [31, 32]. GFRA1-PBD demonstrated synthetic lethality with DDR deficiency, both *in vitro* in BRCA1-isogenic paired cells and *in vivo* in the CTG-0012 model (Supplementary Figure 5 and Figure 5B), suggesting enhanced sensitivity of tumor cells to a GFRA1 ADC despite low receptor density. Although CTG-0012 displayed the lowest level of GFRA1 expression in the study, we still observed tumor regression at 1 mg/kg and tumor stasis at a lower dose of 0.3 mg/kg. This phenomenon could arise from a compromised ability in the model to repair DNA double-strand breaks due to inactivating mutations in BRCA1, ATM, BLM, and p53 [33]. All other models displayed no known defects in DDR. Our data therefore suggest that target-positive, DDR-defective tumors might be hypersensitive to PBD-based ADCs. This hypersensitivity could be a powerful mechanism by which to increase the therapeutic potential of DNA-damaging ADCs, but more study is needed. Future work should include a larger number of weak expressing GFRA1 models as well as an expanded panel of models with varied DDR deficiencies in order to elaborate upon this synthetic lethality between GFRA1 inhibition and DDR deficiency.

To date, only the RET-ADCs Y078- DM1 and Y078-DM4 have been described as targeting a similar pathway to GFRA1-mediated inhibition in breast cancer [34]. Although GFRA1 and RET appear to function together in a canonical signaling setting downstream of GDNF, several labs have described the RET-independent expression and internalization of GFRA1 [9, 14, 35]. Taken together, these data suggest that targeting GFRA1 could have an impact on different tumor subsets, with unique toxicity and activity profiles compared with RET. Specifically, our data propose that targeting GFRA1 could have some specific advantages. First, GFRA1 overexpression is nearly twice as prevalent as RET, as was shown by a detailed IHC analysis in 245 breast cancers [14]; thus, targeting GFRA1 could reach a wider range of breast cancers. Second, GFRA1 expression in normal tissue is often distinct from and more limited than RET, particularly in the central and peripheral nervous systems [11, 36–38], and on-target toxicity could be reduced. Indeed, our paired-rat normal-tissue expression and toxicity studies did not reveal any signs of on-target toxicities in GFRA1 expressing organs (Figure 6 and Supplementary Figure 6). Interestingly, a recent *Nature Medicine* report on a GFRA1 homolog has illustrated the conservation of GFRA1 expression patterns across mouse, rat, cynomolgus monkey, and human [39]. These data support the physiologic relevance of our toxicity studies in rat and further evaluation of the GFRA1-PBD ADC in non-human primates.

Escalating ADC dosages to therapeutic levels in Phase 1 clinical trials while avoiding toxicities is a major challenge that has faced ADC clinical development since its inception. One mechanism to increase the clinical therapeutic index of ADCs pursues antigens that exhibit minimal non-tumor sinks that could affect pharmacokinetics of the ADC and display limited normal-tissue expression that might confer on-target toxicity. GFRA1 exhibits a confined expression profile in critical normal tissues: expression is restricted to normal breast tissue and the central and peripheral nervous systems (Figures 1 and 3A). This expression profile could serve to widen the clinical therapeutic index of a GFRA1 ADC through multiple means. Namely, GFRA1 expression in noncycling, terminally differentiated neuronal cells protected by the blood brain barrier will likely display minimal sensitivity to PBD-based, DNA-damaging payloads. Further, the apparent partial cytoplasmic expression of GFRA1 in normal breast tissues would prevent receptor-mediated cellular uptake of the ADC. However, other factors outside of solely target expression could contribute to ADC on-target toxicity, such as cell cycle kinetics, accessibility to the ADC, dispensability of the target-expressing organ, and it’s regenerative potential [5]. These factors could serve to minimize the potential on-target toxicity that the cell surface expression of GFRA1 in normal breast tissue might imply. As seen in the case of Ado-trastuzumab emtansine, HER2 is a TAA expressed in normal breast tissue, yet the major dose limiting toxicities of this ADC have not been centered around breast, rather thrombocytopenia and elevated liver enzymes [5]. Perhaps breast tissue expression does not necessarily predict eventual on-target ADC toxicity. Taken together, the limited expression profile of GFRA1 in normal tissues, coupled with the biology of the specific GFRA1 expressing organs are critical factors which could serve to widen the therapeutic index of a GFRA1- targeted ADC.

Conversely, one could envision that peripheral nerve expression of GFRA1 has the potential to lead to issues of toxicity, particularly among heavily pretreated cancer patients who often suffer from preexisting peripheral neuropathy [40]. However, there is precedence for the safety and tolerability of clinical-stage, antibody-based therapeutics targeting proteins expressed in peripheral nerve. IMGN901 targets the glycoprotein CD56 (NCAM1), which is known to be expressed widely in the central and peripheral nervous systems [41]. A phase II trial assessing this construct was recently discontinued, but this was largely attributed to a lack of efficacy and infection, rather than dose-limiting neural toxicity. Another example of a TAA with neural expression, GD2, has been clinically tested as a radiolabeled antibody, cytokine fusion, and chimeric antigen receptor. Pain has been associated with IgG-based GD2 therapies, but not with the chimeric antigen receptor, suggesting that this toxicity may be related to the construct and not the target [42]. Additionally, the selection of a DNA-targeting payload on the GFRA1 ADC may offer a mechanism to minimize the risk of peripheral neuropathy. To this end, clinical data has revealed that patients treated with auristatin, maytansinoid, and taxane antibody drug- conjugates often display cumulative and dose-limiting peripheral neuropathy, whereas such toxicities are not associated with PBD based ADC’s [43]. Because there is a lack of predictive preclinical models of peripheral neuropathy [44], it is challenging to understand if there could be a collective impact of both target-driven and payload-driven peripheral neuropathy prior to clinical testing. Interestingly, a preclinical GFRA1-vc-MMAE ADC has recently been described, thus further development of this compound could help elucidate the causes of ADC-mediated nerve toxicity [19].

In summary, our work has demonstrated that GFRA1 can serve as a TAA in multiple subsets of breast cancer. We have engineered a GFRA1-targeted ADC that demonstrates activity in PDX models encompassing a range of heterogeneity in GFRA1 expression. This ADC was cross-reactive with rat GFRA1 and did not show any potential for on-target toxicity in rat toxicity studies. Our data support further preclinical development of this PBD-based GFRA1-targeted ADC.

## MATERIALS AND METHODS

### Cell lines and transfection

Cells were obtained from American Type Culture Collection (Manassas, VA) and grown according to their recommendations. Cells were grown at 37°C and 5% carbon dioxide in a humidified incubator. Cell-line authentication was conducted by short tandem repeat-based DNA-fingerprinting and multiplex PCR, and the absence of mycoplasma was verified. To ensure cells would be at a similar passage for all experiments, cells were cultured only a few passages and banked. Human perineurial cells and rat dorsal root ganglia cells were purchased from ScienCell Research Laboratories (Carlsbad, CA), cultured according to their recommendations and not passaged. MCF10a BRCA1-positive and clustered, regularly interspaced short palindromic repeat (CRISPR)-negative cells were purchased from Horizon Discovery (Cambridge, United Kingdom) and cultured according to the manufacturer’s recommendations. Because MCF10a cells do not express GFRA1, the BRCA1-positive, CRISPR-negative MCF10a cell lines were transduced with lentivirus encoding GFRA1 to create genetically defined models. Stable Ad293 cells expressing green fluorescent protein (GFP), phycoerythrin (PE), or human, murine, rat, or cynomolgus monkey GFRA1 were created using lentiviral expression vectors produced through the pPACKH1-XL packaging mix (System Biosciences, Mountain View CA), followed by transduction and puromycin selection. Transfection of On-Target plus Smart Pool small interfering RNA (siRNA) reagents (GE Dharmacon, Lafayette CO) was carried out using RNAiMax (Thermo Fisher Scientific, Waltham, MA) according to the manufacturer’s instructions. Cells were assayed within 72 h of transfection.

### Generation and characterization of anti-GFRA1 antibodies

GFRA1 antibodies were generated at Akesobio (Guangdong, China). Mice were immunized with a His-tagged GFRA1 extracellular domain purified protein over 4 weeks. Following two boosts, spleens and lymph nodes were harvested from the mice before B-cell fusion with myeloma. The serum titer was evaluated by ELISA using a GFRA1-human Fc screening reagent. Myeloma fusion was performed in 20 by 96 well plates. Following cloning and sequencing, recombinant immunoglobulin G (IgG) molecules were generated and transferred to MedImmune for incorporation into an ADC IgG backbone Clones 4D12, 10H9, 9B3, and 18B2 were used in this study. To minimize cross-reactivity to closely related family members, hybridomas were screened by GFRA1 and GFRA2 ELISA and by fluorescence-activated cell sorting (FACS) in ACHN and MCF7 cells, which endogenously express GFRA1; in Ad293 cells that stably overexpressed human, mouse, rat, or cynomolgus monkey GFRA1 proteins; and in ACHN cells transfected with GFRA1 siRNA.

ADCs were generated by site-specific conjugation of tesirine (SG3249) to GFRA1-binding IgG and non-binding control IgG. Both IgG’s comprised a cysteine inserted at position 239 in the antibody framework and tesirine was attached via thiol-maleimide coupling as previously described [45, 46]. ADCs were characterized by mass spectrometry and size exclusion chromatography to determine drug load and percent monomer.

#### FACS

FACS was performed by harvesting approximately 80% confluent cell cultures using Cell Dissociation Buffer (Thermo Fisher Scientific, Waltham, MA) and transferring cells into 96-well plates for staining. Cells were incubated at 4^○^C with anti-GFRA1 antibodies for 1 h and with secondary anti-human fluorescein isothiocynate (FITC) antibodies (Thermo Fisher Scientific, Waltham, MA, USA) for 30 min. Cells were stained with 4′,6-diamidino-2-phenylindole (DAPI), analyzed on a MACS Quant Flow Cytometer (Miltenyi Biotec Inc., San Diego, CA) for FITC positivity. Mean fluorescence intensity (MFI) values from a nonspecific control IgG1 were subtracted as background.

#### Internalization assay

Clones were further triaged through an internalization assay that was performed by conjugating control IgG and 10H9 with pHrodo Green STP ester pH-sensitive dye according to the manufacturer’s instructions (Thermo Fisher Scientific, Waltham, MA). Cells were also labeled with Cell Tracker Red (Thermo Fisher, Waltham, MA). Green and red fluorescence signals, as well as bright-field imaging, were monitored by Cellomics Array Scan (Thermo Fisher Scientific, Waltham, MA) image acquisition at 37°C every 30 min for 5 h. Data was plotted as the percentage of cells containing internalized antibody.

#### IHC

IHC staining of formalin-fixed, paraffin-embedded samples was performed on a Ventana Discover ULTRA instrument (Ventana Medical Systems, Tucson, AZ, USA) using anti-GFRA1 antibody 4D12. Briefly, samples were treated with Pretreatment Enhanced Cell Conditioning for 48 min at 95^○^C, Discovery inhibitor for 12 min, and primary/isotype antibody at 1.5 mg/ml for 16 min at 35^○^C. Subsequently, samples were subjected to linking antibody treatment at 2 mg/ml for 16 min, secondary OmniMap-HRP for 12 min, ChromoMab DAB for 4 min, and finally hematoxylin and bluing reagent. Slides were imaged on the Aperio Slide scanner (Leica Biosystems Inc., Buffalo Grove, IL). Grading of staining was done at MedImmune according to a scoring system that incorporates intensity and heterogeneity: weak (any cells with weak membrane staining, but less than 50% that are moderate/strong), moderate (more than 50% of the cells display moderate staining, but less than 50% with strong staining) strong (more than 50% of the cells displaying strong staining). Similarly, optimized Ventana protocols were also utilized for rat GFRA1 (AF560, R&D Systems, Minneapolis, MN), γH2A.X Ser 139 IHC (9718, Cell Signaling Technology, Danvers, MA) and SG3199 (276-A8, Biogenes GmbH, Berlin, Germany). All normal and tumor microarrays were purchased from US Biomax (US Biomax, Inc., Rockville, MD).

### Determination of receptor density

GFRA1 receptor density was determined by FACS, using the anti-GFRA1 clone 10H9 and the Quantum Simply Cellular anti-human kit (Bang’s Laboratories, Inc., Fishers, IN, USA) according to the manufacturer’s instructions. In short, cells were harvested as above for FACS, but counted and seeded 200,000 cells per well in 200 ml buffer. Cells were incubated with 10 mg/ml 10H9 that had been conjugated to GFP using the Alexa Fluor 488 Antibody Labeling kit (Thermo Fisher Scientific, Waltham, MA). Samples were analyzed on a MACS Quant Flow Cytometer (Miltenyi Biotec Inc., San Diego, CA) for MFI of the GFP positive population. To assign antibody binding capacity, a standard curve was generated using a bead-dilution series included in the kit.

#### Quantitative real-time-PCR

The Oncomine Power Tools database was used to assess *GFRA1* mRNA expression patterns in cancer and normal tissues. cDNA arrays with 48 normal tissues or 92 breast cancer tissues (Origene, Rockville, MD, USA) were probed for GFRA1 and glyceraldehyde-3-phosphate dehydrogenase (GAPDH) mRNA levels with the Fluidigm system (San Francisco, CA, USA). cDNA was pre-amplified with Taqman gene-specific probes (Invitrogen, Carlsbad, CA) before quantitative PCR. Data were normalized to GAPDH and made relative using 2−ΔCt method.

#### Sandwich ELISA and octet

To assess the level of soluble GFRA1 antigen, a standard sandwich ELISA was performed with two anti-GFRA1 antibodies to different epitopes: 9B3 as a capture antibody and 18B2 as a detection antibody. Briefly, Nunc Maxisorp plates (Thermo Fisher Scientific, Waltham, MA, USA) were coated with 5 mg/ml 9B3 antibody at 4^○^C overnight, washed, and blocked with 1% bovine serum albumin (Sigma-Aldrich, St Louis, MO). Human serum samples from Conversant Bio (Huntsville, AL) or from cell-line-conditioned media (normalized to cell number) were added to plates, along with recombinant human GFRA1 protein (Sino Biological, Beijing, China) as a positive control. Samples were incubated for 30 min at room temperature before washed and biotinylated 18B2 added for subsequent incubation with streptavidin-horseradish peroxidase. Soluble GFRA1 was detected on a Spectramax plate reader (Molecular Devices, Sunnyvale, CA) at 450 nM and quantified using a standard curve. Antibody-affinity measurements were gathered using Octet (Pall ForteBio, Fremont CA), where antibodies were immobilized on anti-hFc biosensor, and association and dissociation rates of human, mouse, rat and cynomolgus recombinant protein (Sino Biologicals, Beijing, China) were assessed to generate dissociation constants (Koff/Kon).

### *In vitro* cytotoxicity assay

The cytotoxicity of the 10H9 clone conjugated to the PBD SG3249 was evaluated *in vitro* against a panel of cell lines. Cells were plated in culture media at 2,000 or 4,000 cells per well (depending on cell-line growth kinetics) of a tissue culture-treated, 96-well plate. Cells were plated in 80 µL of media and allowed to grow overnight. Treatments were prepared at five times stock concentration in culture medium, and 20 ml was added to cells. Cells were treated in triplicate in a dose range starting at a high concentration of 10 ug/ml and diluted stepwise in a 1:10 fashion to 0.01 pg/ml. Treated cells were cultured for 120 hours at 37°C and 5% carbon dioxide before viability was determined using the CellTiter-Glo^®^(CTG) Luminescent Viability Assay (Promega Corporation, Madison, WI) according to the manufacturer’s instructions. Absorbance was read at 560 nM on an EnVision luminometer (Perkin Elmer, Waltham, MA), and raw values were used to calculate percent cell viability (average luminescence of treated samples/average luminescence of control samples) × 100. GraphPad Prism software was used to determine IC_50_ values via logistic non-linear regression analyses. The Caspase-Glo 3/7 Assay was used according to the manufacturer’s instructions (Promega, Corporation, Madison, WI), and data was displayed as a ratio of Caspase 3/7 values to CellTiter-Glo luminescence readings.

### Assessment of bystander activity elicited by a GFRA1 ADC

Ad293 cells transduced with PE- or GFRA1-FITC-encoding lentivirus were co-cultured with increasing doses of 10H9-SG3249 (GFRA1-PBD) or nonspecific IgG1-SG3249 (Control-PBD) for 6 days, then subjected to flow cytometry.

### *In vivo* studies of ADC activity

All *in vivo* studies were carried out in compliance with American Association for Assessment and Accreditation of Laboratory Care (AALAC) guidelines and according to MedImmune Institutional Animal Care and Use Committee (IACUC) approval. Cell–line-derived xenograft models were developed by injecting a 1:1 ratio of tumor cell line and Matrigel mixture subcutaneously into the flanks of 5- to 6-week-old athymic nude mice (Envigo). Resulting tumors were measured twice per week, and tumor volume was calculated by using the formula: tumor volume (mm^3^) = (length × width^2^)/2. Mice were randomized when tumor volumes had reached 200 mm^3^, and ADCs were administered at 1 mg/kg once intravenously based on pilot studies. Tumor growth was monitored at least twice weekly.

PDX models were chosen from contract research organizations based on GFRA1 gene expression and IHC analysis. *In vivo* studies of ADC activity were carried out in PDX models at Xentech Evry, France), Champions Oncology (Hackensack, NJ), or Horizon Discovery (Saint Louis, MO) according to standard procedures. PDX models were grouped based on the level of GFRA1 expression, as determined by IHC scoring of PDX tumor sections, and assessed as described above for the other xenograft models. PDX models were from different tumor origins: Breast cancer (WHIM16, WHIM18, T226, CTG-0012, CTG-0017), Ovarian cancer (CTG-0258, CTG-0257, CTG-0992, CTG-0897). Tests for synthetic lethality between GFRA1 inhibition and DNA damage-repair (DDR) deficiencies were tested at a standard ADC dose of 1 mg/kg or a low dose of 0.3 mg/kg.

### Rat toxicology study

The rat study was conducted at a facility that complies with the principles of the ‘Guide for Care and Use of Laboratory Animals’ and is accredited by the Association for Assessment and Accreditation of Laboratory Animal Care International (AAALAC). Study protocol was approved by the testing facilities IACUC. Male Sprague Dawley rats were administered a single intravenous injection of vehicle control (10 animals), 0.75 mg/kg (16 animals), or 1.5 mg/kg (16 animals) anti-GFRA1 conjugated to SG3249 on Day 1. Animals were necropsied on Days 8 and 22 (five animals per time point) to evaluate the acute and delayed effects of the test article. Animals were evaluated for clinical signs, body-weight changes, clinical pathology, gross pathology with organ weights, and histopathological examination by a board-certified veterinarian pathologist. Hematology, coagulation, and serum chemistry samples were collected on Days 8, 15, and 22. Additionally, six animals from the 0.75 mg/kg and 1.5 mg/kg dosing groups were utilized for toxicokinetic monitoring of the plasma concentration of total antibody on Days 1, 2, 4, 8, 15, and 22. Total ADC in rat sera was quantified by sandwich ELISA (as described above) by using SG3199 276-A8 as the capture antibody bound to the plate, and a goat anti human IgG – HRP conjugated antibody (Bethyl Laboratories, Inc, Montgomery, Texas) was used for detection.

## SUPPLEMENTARY MATERIALS FIGURES AND TABLE


