# Supplementary material for: Preclinical evaluation of a GFRA1 targeted antibody-drug conjugate in breast cancer
Source: Oncotarget. 2018 May 1;9(33):22960–75. doi: 10.18632/oncotarget.25160 (PMC5955426; doi:10.18632/oncotarget.25160)
Supplement: Supplementary file 1 [file oncotarget-09-22960-s001.pdf]

Preclinical evaluation of a GFRA1 targeted antibody-drug conjugate in breast cancer

SUPPLEMENTARY MATERIALS

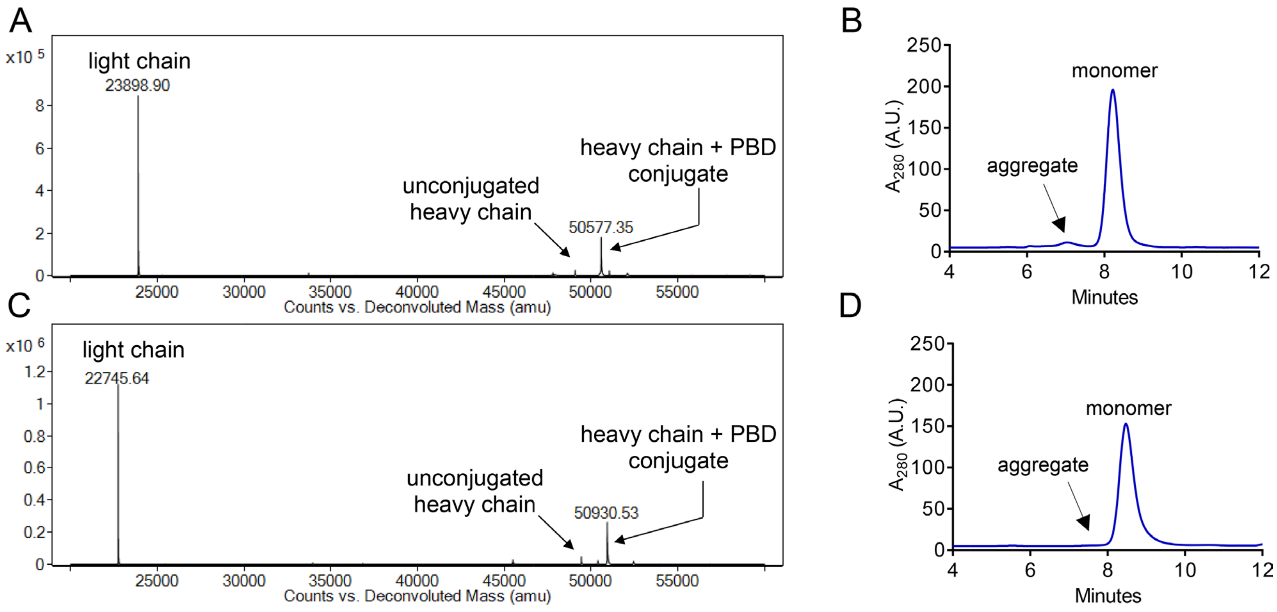

E Summary of ADC Characterization Data

ADC	Drugs per antibody	% monomer
GFRA1-PBD	1.8	98
Control-PBD	1.7	99

Supplementary Figure 1: Characterization of ADC's. (**A**) Determination of drug content in GFRA1-PBD ADC by reduced deglycosylated mass spectrometry analysis. (**B**) Aggregate analysis in GFRA-PBD ADC by size exclusion chromatography. (**C**) Determination of drug content in Control-PBD ADC by reduced deglycosylated mass spectrometry analysis. (**D**) Aggregate determination in Control-PBD ADC by size exclusion chromatography. (**E**) Summary of the GFRA1-PBD and Control-PBD drug antibody ratios and monomer content. PBD, pyrrolobenzodiazepine.

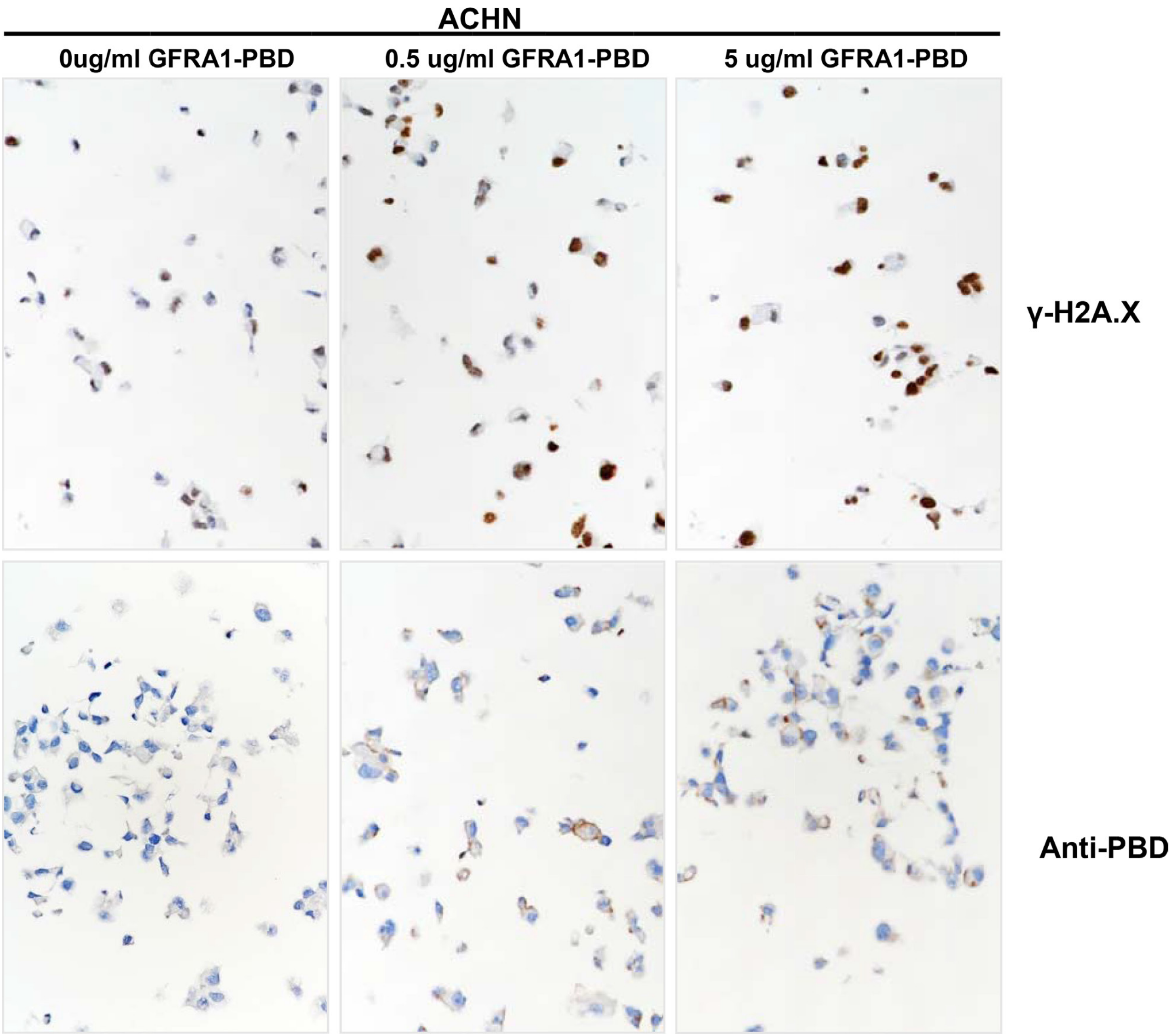

Supplementary Figure 2: ACHN cells treated with 0, 0.5, or 5 µg/ml SG3249-10H9 or SG3249-control were formalin fixed, paraffin embedded, and pelleted 24 h following treatment. Cell pellets were stained by using an optimized Ventana protocol for both the anti-PBD antibody and γH2A.X, compared with isotype control. γH2A.X, phosphorylated H2A histone family member X.

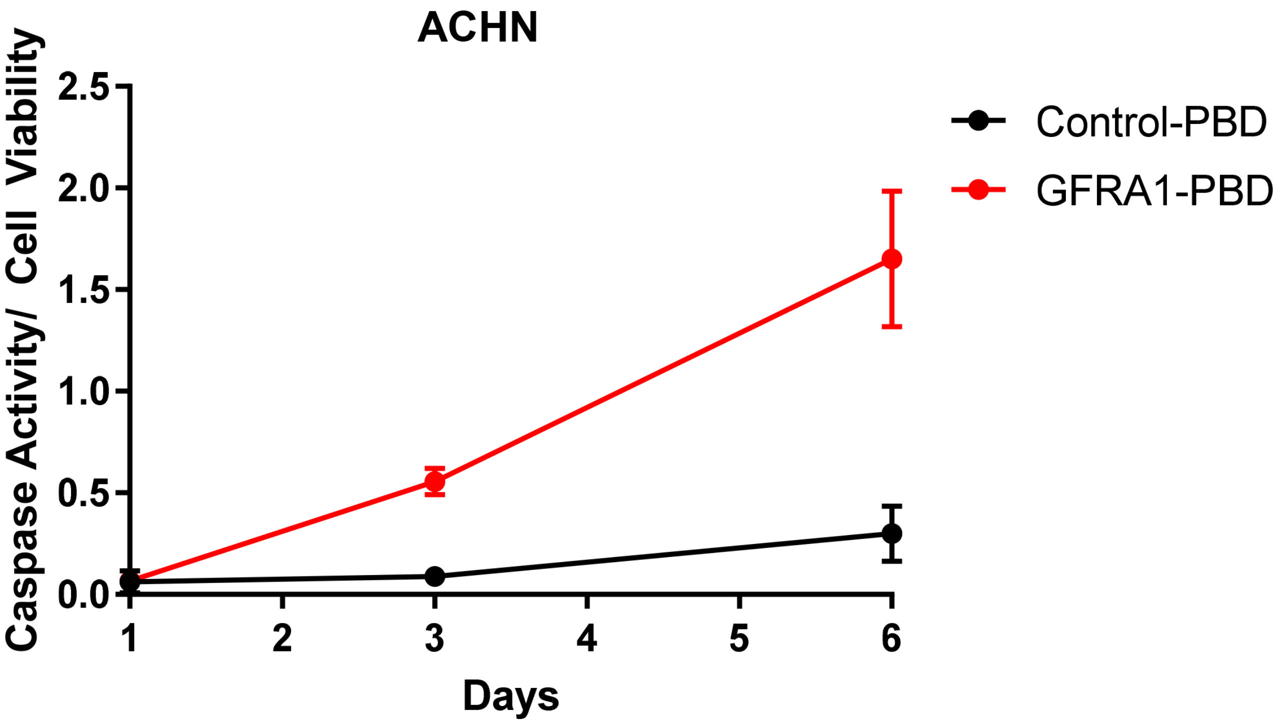

Supplementary Figure 3: ACHN cells were treated with 1 µg/ml of either GFRA1-PBD or control-PBD, and the levels of caspases 3 and 7 were determined.

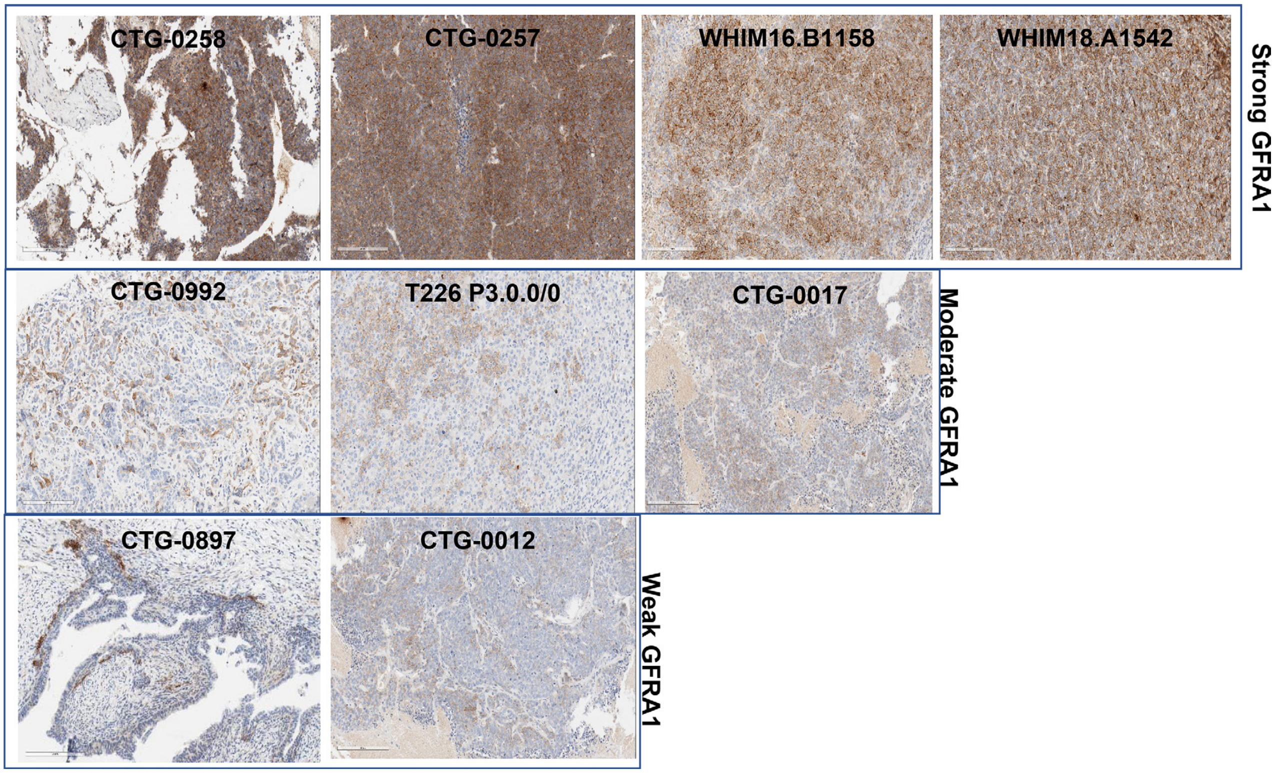

Supplementary Figure 4: GFRA1 IHC was performed in tissue sections from PDX models used in *in vivo* efficacy studies. Staining pattern was graded based on GFRA1 membrane intensity and heterogeneity.

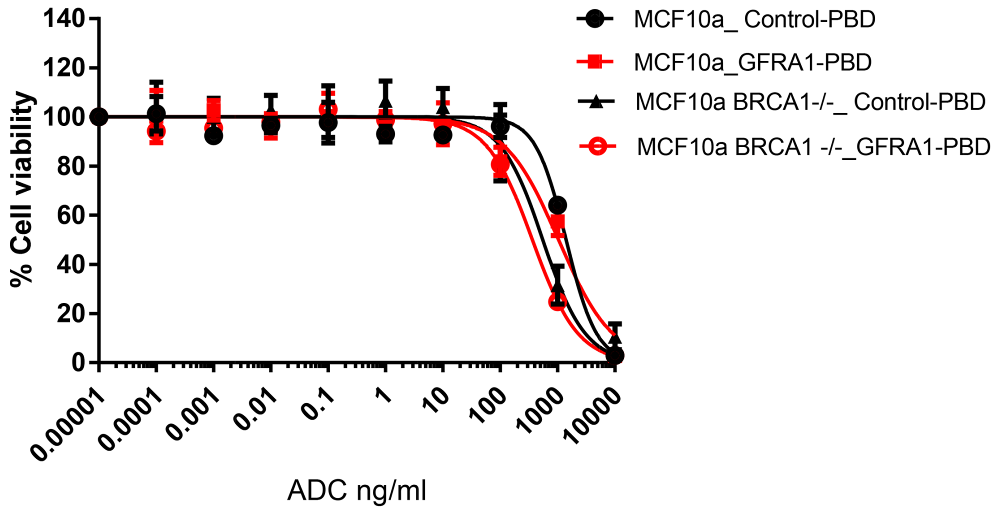

IC50 (ng/ml)	Max Kill (%)
-	-
-	-
-	-
142	98

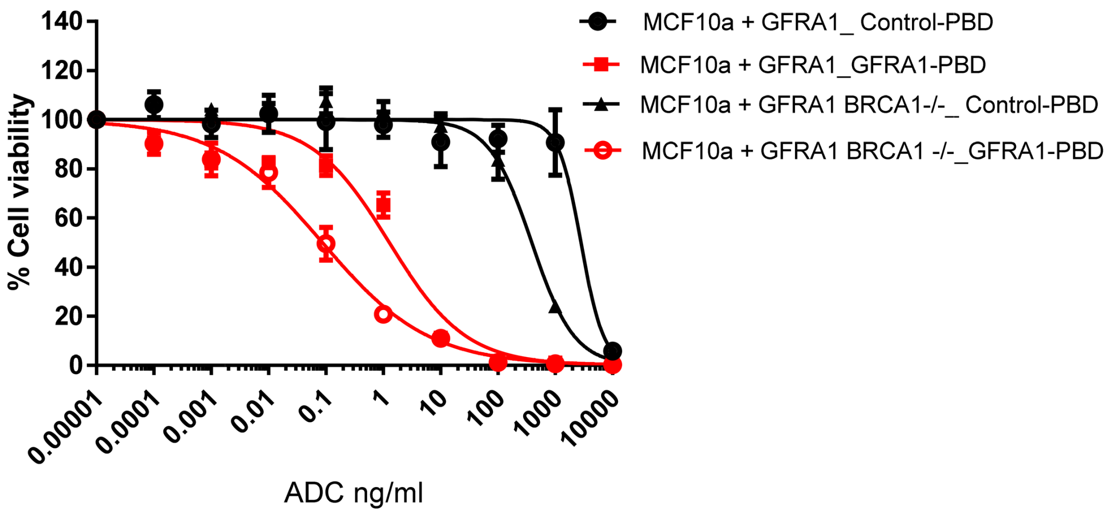

IC50 (ng/ml)	Max Kill (%)
2818	95
1.276	99
389	95
.087	99

Supplementary Figure 5: MCF10a cells that were BRCA1-proficient or deficient (via CRISPR, Horizon Discovery) were treated with Control-PBD or GFRA1-PBD in a 6 day viability assay (top panel). Both cell types were transduced with GFRA1 and again treated with Control-PBD or GFRA1-PBD in a 6 day viability assay (bottom panel).

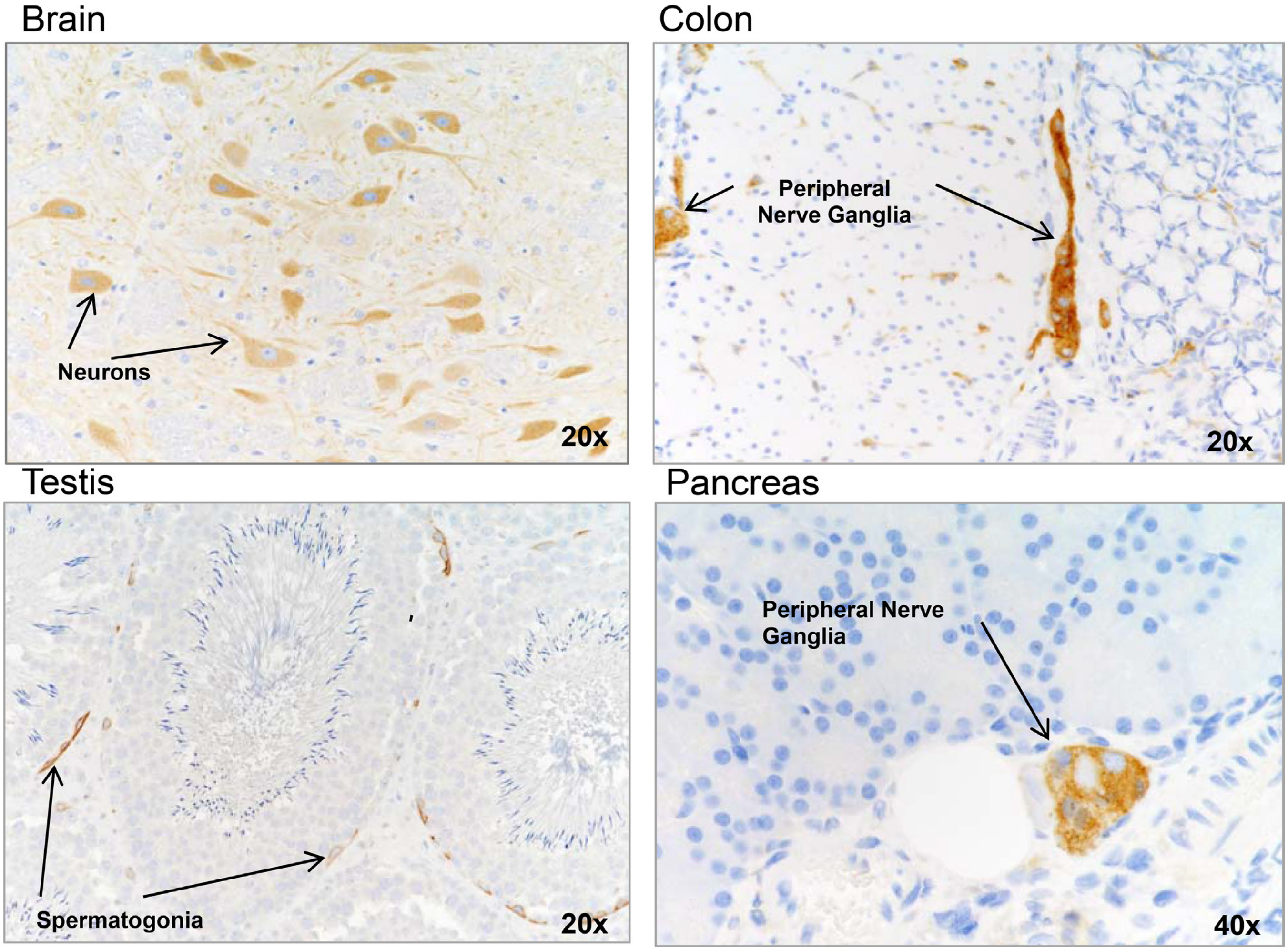

Supplementary Figure 6: IHC was performed on large tissue sections of rat brain, colon, testis, and pancreas for rat GFRA1 expression, and specific membrane positive cell types are highlighted. All isotype control sections were negative, as well as all other normal rat tissues from a normal rat tissue TMA (901a, US Biomax, Gaithersburg, MD).

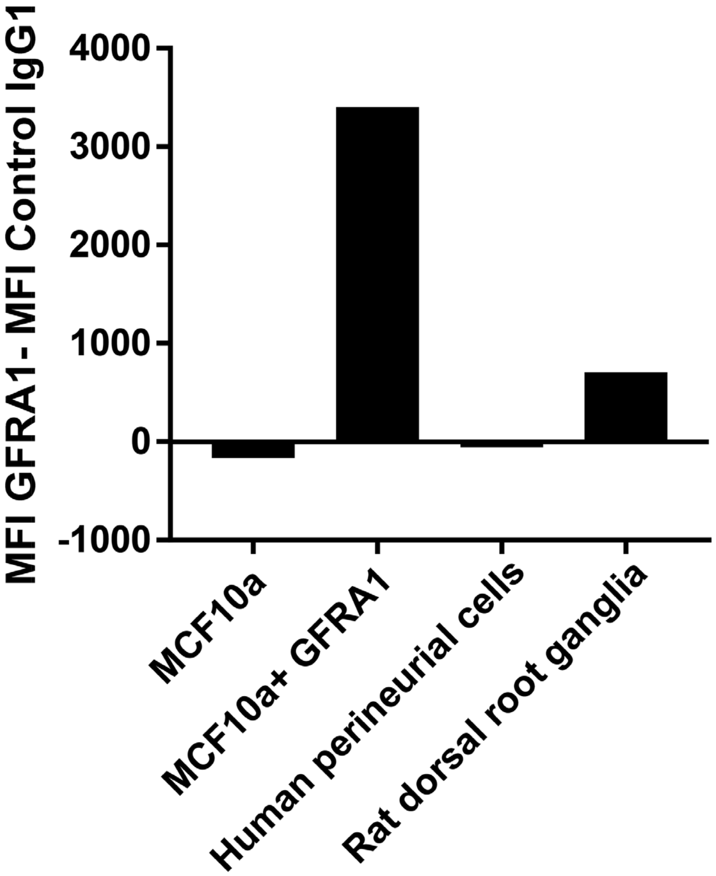

Supplementary Figure 7: FACS analysis of GFRA1 10H9 clone binding was performed on MCF10a human mammary epithelial cells, MCF10a cells transduced with lentiviral GFRA1, human perineurial primary cells, and rat dorsal root ganglia cells. Data is displayed as MFI of 10H9 minus the MFI of the nonspecific IgG1 control.

Supplementary Table 1: Anti-GFRA1 antibody characteristics

	mAb Characteristics			
	**mAb**	**Antigen**	**KD (nM)**	**IC50 (ng/ml)**
**Epitope group 1**	**4D12**	Human	6.2	0.89
		Cyno	ND	0.9
		Mouse	9.36	2.7
		Rat	5.97	50
	**9B3**	Human	16.4	9.4
		Cyno	ND	8.9
		Mouse	36.1	70
		Rat	9.40	36
**Epitope group 2**	**10H9**	Human	6.9	2.2
		Cyno	ND	2.1
		Mouse	32	70
		Rat	5.05	75
	**18B2**	Human	1.6	1.1
		Cyno	ND	2.3
		Mouse	6.68	8
		Rat	3.39	7.9

Four anti-GFRA1 antibodies were generated from the screen. Species cross-reactivity, affinity to monomeric antigen using Octet KD measurement, and in vitro cell-kill activity in Ad293-GFRA1 cells upon conjugation to SG3249 are listed for each antibody. A competition experiment was performed via FACS to determine the epitope specificity of the four mAbs. FACS, fluorescence-activated cell sorting; ND; not determined, mAb, monoclonal antibody.
